# Advancing Viscoelastic Material Characterization Through Computer Vision and Robotics: MIRANDA and RELAPP

**DOI:** 10.3390/ma18214827

**Published:** 2025-10-22

**Authors:** Antonio Monleón-Getino, Víctor Madarnás-Gómez, Mario Cobos-Soler, Eduard Almacellas, Juan Ramos-Castro, Xavier Bielsa, Pere López-Brosa, Àngels Sahuquillo-Estrugo, Inés Marsà-González, Alejandro Rodríguez-Mena

**Affiliations:** 1 BIOST3 (Research Group in Biostatistics, Data Science and Bioinformatics), Research Group in Biostatistics, Data Science and Bioinformatics, 08028 Barcelona, Spain; 2 Department of Genetics, Microbiology and Statistics, Universitat de Barcelona, 08028 Barcelona, Spain; 3 Electronic Engineering Department, Universitat Politècnica de Catalunya, 08034 Barcelona, Spain; 4 Laboratory Mat Control, Department of Chemical Engineering and Analytical Chemistry, Faculty of Chemistry, Universitat de Barcelona, 08028 Barcelona, Spain

**Keywords:** robotics, computer vision, viscoelastic material, Chopin alveograph, material characterization, viscoelasticity, viscosity

## Abstract

This study introduces MIRANDA, a computer vision system, and RELAPP, a complementary force measurement system, developed for characterizing viscoelastic materials. Our aim was to evaluate their combined ability to predict key rheological parameters and demonstrate their utility in material analysis, offering an alternative to traditional methods. We analyzed five distinct flour dough samples, correlating MIRANDA and RELAPP variables with established rheological reference values. Support Vector Machine (SVM) regression models were trained using MIRANDA’s stable TR and elasticity data to predict industrially relevant parameters: baking strength (W), tenacity (P), extensibility (L), and final viscosity (RVU) from Chopin alveograph and viscosimeter. The predictive models showed promising results, with R^2^ values of 0.594 (*p* = 0) for W, 0.575 (*p* = 0) for P, and 0.612 (*p* = 0.03763) for viscosity, all statistically significant. While these findings are promising, it is important to note that the small sample size may limit the generalizability of these models. The synergy between the systems was evident, exemplified by strong positive correlations, such as between MIRANDA’s Elasticity and RELAPP’s c_exp (parameter ‘c’ of its mathematical model m1, r = 0.858) and final resistive force (r = 0.839). Despite the limited sample size, these findings highlight MIRANDA’s versatility and speed for efficient material characterization. MIRANDA and RELAPP offer significant industrial implications for viscoelastic materials, including accelerating development cycles and enhancing continuous quality control. This approach has strong potential to reduce reliance on slower, traditional methods, warranting further validation with larger datasets.

## 1. Introduction

Viscoelasticity is a fundamental physical property inherent to a large quantity of materials, ranging from synthetic polymers to biological tissues and numerous food products. These materials, which combine properties of ideal elastic solids and viscous fluids, are characterized by distinct behaviors that often depend on time and serve as fundamental identifying characteristics. Even minor modifications in these properties can significantly influence their functionality or use. In the food industry, for instance, the rheological, and particularly the viscoelastic, properties of products such as doughs, gels, or emulsions, determine critical aspects like texture, palatability, storage stability, and ultimately, their industrial performance [1,2]. The precise characterization of these properties is key for quality control in manufacturing and for the development of new products.

Traditionally, rheological analysis is carried out using specialized instruments such as rheometers, texturometers, Chopin Alveographs, Rapid Visco Analyser (RVA) and Brabender Consistographs [3,4,5]. Chopin Alveographs are particularly crucial in the flour dough and derivatives industry [6], where they provide quantitative measures of dough strength, elasticity, and consistency under controlled conditions [7,8,9]. Similarly, the Rapid Visco Analyser (RVA) evaluates the viscosity properties of flour starch during gelatinization and retrogradation, measuring key parameters such as peak and final viscosity. However, these conventional methods can be costly, often require specific sample preparation, and are not always easily implementable in production environments for continuous monitoring. In this context, alternative techniques based on computer vision and machine learning are emerging as viable solutions [10,11,12].

This study addresses the critical novelty gap in existing methodologies by introducing a unique dual-system approach that combines a passive computer vision system (MIRANDA) with an active robotic rheometer (RELAPP). This unique synergy offers a non-invasive, cost-effective, and highly repeatable platform for comprehensive material characterization, overcoming the limitations of conventional rheological instruments.

While prior work, such as that by [13,14], has explored the use of force and displacement, our approach innovates by using computer vision to objectively quantify a material’s non-contact deformation and recovery phase, which significantly reduces the subjectivity inherent in manual or single-point measurements.

Recent research has focused on developing more sophisticated constitutive models that account for rate-dependent effects and complex material behaviors [15].

### Theoretical Background

Understanding the deformation behavior of soft materials necessitates a theoretical framework rooted in continuum mechanics and rheology. We conceptualize the material as a continuous medium, allowing the application of calculus tools to describe its state under external forces. This theoretical foundation is explored in much greater detail than presented here in the Bachelor’s thesis by Almacellas-Canals [13], titled “Classification of viscoelastic materials based on rheological parameters estimated using computer vision and deep learning techniques.” The theoretical models and mathematical justifications used for the creep and recovery phases are summarized in Appendix A.

## 2. Materials and Methods

Addressing the limitations of current material characterization methods, a novel system named MIRANDA (Measuring Intelligent Robotic Analysis Network Developed Around) has been developed. This innovation signifies a paradigm shift in material science, offering a non-invasive platform for detailed viscoelastic analysis and motion dynamics. MIRANDA leverages a unique combination of computer vision and advanced algorithms to provide real-time, non-invasive insights into material deformation and movement. This innovative approach effectively simulates the nuanced perception of human touch, delivering unprecedented analytical capabilities in a portable and cost-effective device. MIRANDA is designed for the viscoelasticity measurement of various materials, including flour doughs, pastry, foams, soft plastics, and silicones.

**Figure 1:**
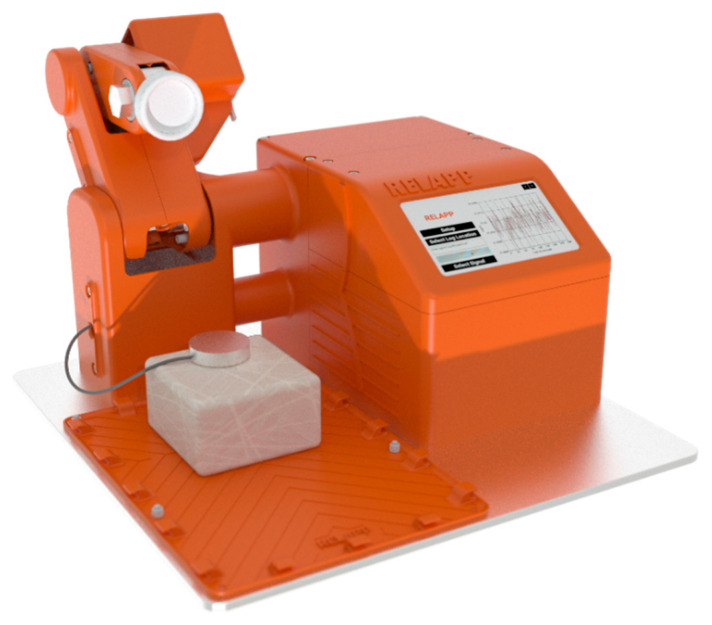
The RELAPP Robot, with its various degrees of freedom (DOF), enables controlled material deformation and subsequent observation. It is equipped with a sensor that collects data from the material during the test, making it a valuable tool that complements systems like MIRANDA (computer vision analysis) for analyzing the viscoelastic properties of materials, specifically through Creep-Recovery Test (See Appendix A).

Complementing MIRANDA’s passive, vision-based approach is RELAPP (Rheometry APP), a sophisticated, portable, and low-cost robotic rheometer (Figure 1). RELAPP emerged from a previous project where a simpler robotic deformer and soft material analyzer was developed, published as “RELAPP: A New Portable Electronic Rheometer for the Analysis of Viscoelastic Materials Based on Artificial Intelligence” [14], demonstrating a reasonable prediction of flour dough variables typically measured with the Chopin Alveograph.

RELAPP utilizes a multi-degree-of-freedom robotic arm to actively deform soft materials and observe their precise, real-time response through integrated sensors. This active deformation capability allows for accurate measurement of material response to controlled forces, providing insights into industrial rheological parameters such as tenacity, viscosity, extensibility, and consistency across a diverse range of soft materials. Together, MIRANDA and RELAPP represent a powerful duo for comprehensive viscoelastic characterization, offering versatile solutions where traditional bulky and costly equipment is impractical. The robot control and data acquisition processes are managed by microcontrollers, which are synchronized and coordinated through a Raspberry Pi. This Raspberry Pi enables workflow streamlining, as well as real-time data processing and visualization of the collected data through a custom-built, user-friendly touch-based GUI application.

This work addresses the development and application of a system based on computer vision and deep learning techniques that allows obtaining key characteristics of viscoelastic materials subjected to controlled deformations through the application of forces, analyzing the initial deformation and its subsequent recovery (creep-recovery). The main objective is to estimate parameters that enable the characterization of the rheological properties of materials by monitoring their superficial deformation over time, as recorded in video [13]. To test the capabilities of these developed methods, as well as to ascertain their repeatability, accuracy, and real-world industrial applicability, experiments have been conducted with various soft materials.

### 2.1. Proof-of-Concept Tests

The primary objective of this research is to evaluate the performance, reliability, and complementary capabilities of the MIRANDA software and the RELAPP robotic rheometer in characterizing the viscoelastic properties of various materials. Specifically, this study aims to:

- Demonstrate MIRANDA’s ability to provide real-time, non-invasive analysis of viscoelastic properties and motion dynamics.
- Assess RELAPP’s precision in actively deforming materials and accurately measuring their real-time rheological response.
- Compare the analytical insights provided by both MIRANDA and RELAPP, highlighting their synergistic application for a comprehensive material characterization.
- Validate the portability, cost-effectiveness, and operational efficiency of both devices as next-generation solutions for viscoelastic measurement and motion analysis.

To provide initial evidence of their efficacy, MIRANDA and RELAPP were evaluated in a series of proof-of-concept tests. These experiments were conducted using a diverse range of readily available materials, which included substances with well-characterized industrial and physical properties. This selection was chosen to represent varying degrees of elasticity and plasticity, enabling a robust assessment of the devices’ performance across material behaviors relevant to industrial applications.

### 2.2. Materials Tested

**Figure 2:**
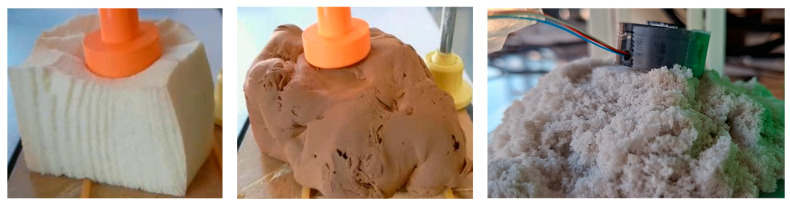
Three examples of materials used for testing with RELAPP and MIRANDA ((**left**) to (**right**): white foam, brown putty and kinetic sand).

A total of 17 distinct material types were analyzed (in Figure 2, some examples are presented). For each material, multiple samples were prepared from 113 observations, and several replicates of the Creep-Recovery Test were performed to ensure the reliability and reproducibility of the results. The materials included:

- Foams: Black, Yellow, Green, and White Foams, representing highly elastic materials.
- Doughs and Pastes: Soft Dough (standard, yellow, white), various Flour Doughs (W-values 90, 119, 262, 400, 461), White Modeling Clay, and Stelan Flexible Paste, representing a range of plasto-visco-elastic behaviors.
- Plasticine and Sand: Plasticine and kinetic sand, to assess plastic and plasto-visco-elastic properties.
- Viscoelastic Materials: A specific Viscoelastic Material and a soft toy made of wheat flour.
- Rigid and Other: Wood (as a rigid reference) and a human hand.

Analyses of the rheological properties of various materials have been conducted using a combination of MIRANDA, RELAPP, and parallel operation of both systems. These studies incorporated different types of replicates, including those performed between distinct materials and those conducted within the same material, to ensure comprehensive and robust data collection.

The selected materials, including specific foams and doughs with varying W-values, were chosen to represent a wide range of viscoelastic behaviors. This allowed us to not only validate the system’s ability to measure diverse materials but also to demonstrate its capacity to predict industrially relevant parameters specific to certain product categories, such as the W-value for flour doughs.

### 2.3. MIRANDA Experimental Setup and Data Acquisition

For MIRANDA, the experimental setup involved a high-speed camera system capturing high-resolution images of the materials. Each sample was subjected to a passive deformation (e.g., a drop or application of a controlled load) or observed during its natural motion dynamics. The core of MIRANDA’s analysis lies in its proprietary algorithms, which process the captured video frames in real time. These algorithms extracted precise data points related to:

- Shape deformation: Changes in the material’s geometric dimensions.
- Texture dynamics: Alterations in surface patterns and visual characteristics.
- Viscoelastic properties: Quantified through “perception units” over time, providing a non-invasive measure of how the material deforms and recovers.

**Figure 3:**
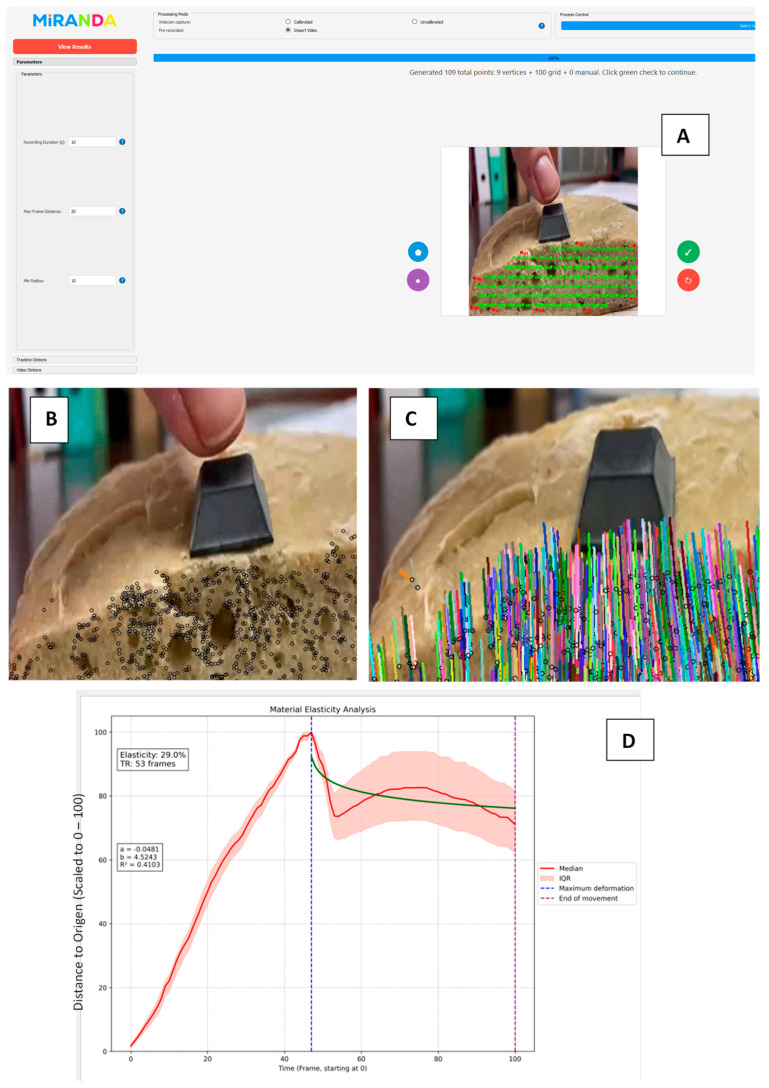
The MIRANDA analysis workflow demonstrated on a rustic bread sample. The process shows the material under observation (**A**), the selection and tracking of key points for analysis (**B**), the observation of the creep-recovery phases (**C**), and the final output curve with calculated parameters (**D**). The green line represents the fit of a 2-parameter mathematical model (m2) to the median data.

For instance, viscoelastic foam (demonstrating nearly 100% elastic behavior) and kinetic sand (approximating 100% plastic behavior) were specifically observed to characterize the extremes of viscoelastic response, with MIRANDA quantifying their distinct “perception units” during the creep-recovery cycles. In Figure 3, the process of analyzing a soft material using MIRANDA is concisely illustrated. This procedure involves observing the material’s movement and deformation under an applied force for a specified duration (e.g., 20 s or as required by the experiment). This deformation can be applied manually or, to ensure optimal repeatability and consistency, a robotic system such as RELAPP can be employed, which exerts a controlled and uniform force over time. This is an example with a complex material (rustic bread): Figure 3A displays MIRANDA’s central interface, showing the rustic bread sample under observation with the specific region of interest highlighted.

Once this deformation is recorded, typically during “Creep-Recovery Test” experiments, key points within the material’s region of interest are identified and observed (as shown in Figure 3, for instance, in rustic bread where the inner crumb is exposed). As shown in Figure 3B, the selected points for analysis are clearly visible, highlighting the bread’s alveoli and the specific locations where the creep-recovery motion trajectories of this elastic material are observed. Subsequently, these points are mathematically analyzed, integrating the physical principles of continuum mechanics and rheology (as discussed in the preceding chapter) with advanced deep learning methods that form the core of MIRANDA. This analysis yields an exceptionally precise description of the median trajectory of the points’ movement, as well as their interquartile variability interval, thereby providing a robust measure of movement dispersion. The creep phase involves a gentle, manual deformation, while the recovery phase is meticulously observed and analyzed using the MIRANDA algorithm (Figure 3C).

From these data, MIRANDA extracts several fundamental material characteristics. These include the material’s median elasticity—defined as the percent of the applied deformation that is recovered the response time (TR)—defined as the time it takes for the material to cease significant movement after load release—and the fit to a specific mathematical model based on the physical principles previously outlined, with a particular focus on the “Recovery” phase of the test. From this fitting process, key parameters such as ‘a’ and ‘b’ are obtained, and the regression coefficient of determination (R^2^) is calculated, which indicates the goodness of fit of the model to the observed data during the recovery phase. The final elasticity analysis curve, along with parameters calculated by MIRANDA, such as the response time and its fit to a specific mathematical model tailored for visco-plasto-elastic materials like baked bread, is presented in Figure 3D.

### 2.4. RELAPP Experimental Setup and Data Acquisition

RELAPP (Figure 1), designed for active rheological measurements, employed a sophisticated multi-degree-of-freedom robotic arm. This arm was programmed to apply controlled, precise deformations to the soft material samples. Integrated sensors within the robotic system captured real-time data on:

- Deformative force: Measured in Newtons (N), indicating the resistance of the material to applied deformation.
- Material displacement/strain: The extent of deformation under the applied force.
- Compactibility: Assessed through successive deformation cycles, providing insights into the material’s ability to withstand repeated stress.

The data acquired by RELAPP, thanks to a system of sensors [14], allowed for the indirect measurement of industrial rheological parameters such as tenacity, viscosity, extensibility, and consistency. These sensors specifically capture the material’s resistance to deformation (which can be interpreted as its tenacity over time during the creep phase) and the material’s movement during the recovery phase. Subsequently, and particularly from the tenacity-time curve, parameters such as the observed maximum force, the fit of the curve to a mathematical model (from which parameters ‘a’ and ‘b’ are obtained), and other derived values are extracted. All data streams from the sensors were synchronized and logged for subsequent detailed analysis.

**Figure 4:**
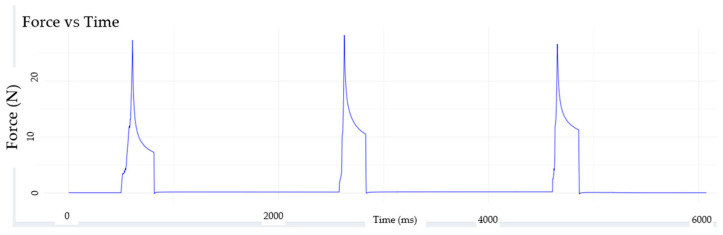
Visualization of the deformation captured by RELAPP sensors for a strength type wheat flour dough (alveographic force, W = 262) during the creep phase using software RELAPP ANALYZER developed to analyze viscoelasticity properties using RELAPP sensor. The *Y*-axis represents the material’s resistive force (N), while the *X*-axis denotes time (milliseconds). This is illustrated in a compactibility test, comparing the undeformed material (**left**) with the material that has been previously deformed twice (**right**) during three Creep-Recovery Tests programmed in the robot.

The RELAPP device offers a robust method for assessing the compatibility and viscoelastic response of materials through the application of repetitive Creep-Recovery Tests. This method allows for a detailed examination of how materials deform under sustained stress (creep phase) and subsequently recover when the stress is removed (recovery phase). Figure 4, for instance, showcases a typical Creep-Recovery Test performed on a strength-type wheat flour dough, specifically with a W (alveographic force) value of 262. This W value is a crucial rheological parameter, commonly measured by a Chopin Alveograph, and indicates a consistent, strong flour highly suitable for breadmaking due to its high gluten content and ability to withstand significant deformation without breaking.

For the visualization and presentation of the deformation curves obtained from RELAPP, the RELAPP Analyzer application (Figure 4), developed by the Biost3 research team, has been utilized. The *Y*-axis represents the material’s resistive force, while the *X*-axis denotes time (milliseconds). This is illustrated in a compactibility test, comparing the undeformed material (left) with the material that has been previously deformed twice (right).

During the initial stages of the test (Figure 4), it is observed that the material undergoes deformation over time, a characteristic behavior of viscoelastic materials. This immediate deformation, followed by continued yielding, clearly demonstrates that the material is not 100% elastic. Instead, it exhibits a combination of viscous (flow-like) and elastic (recovering) properties. The creep phase, where the material deforms under constant load, provides critical insights into its resistance to flow and its ability to store deformation energy.

### 2.5. Data Analysis and Experimental Design

The raw data acquired from both MIRANDA and RELAPP systems underwent comprehensive statistical analysis. The primary goal was to correlate the extracted parameters (e.g., elasticity, force, displacement, perception units) with known rheological properties of the materials. Standard statistical methods were applied to assess the variability, reproducibility, and discriminatory power of the measurements obtained by each device.

To achieve this, an experiment was designed to analyze various common soft and rigid materials, including: elastic foams, putties, the human hand, flour dough, plastic materials, and elastic toys. The design incorporates replicas both within material types and within individual samples. The specific number of replicates (N) for each material type is detailed in the results section, alongside other descriptive statistics, to ensure full transparency regarding the experimental design and data robustness. The presence of missing values (NA or NaN) in the dataset corresponds to parameters that were not applicable for a specific material or were not measured by one of the two systems

Of the materials used, only the physical and rheological properties of the flour doughs are known, utilizing parameters provided by the laboratory where the doughs were prepared at constant hydration. These had been previously determined within the proficiency test scheme that the Laboratory Mat Control organized yearly (Circuito Español de Cereales), with wheat flour and wheat grain quality control materials prepared for this purpose.

Different statistical analyses were performed on the results obtained from both RELAPP and MIRANDA using R and RStudio software. All statistical analyses were conducted using the R programming language (version 4.2.0), with data manipulation and cleaning performed using functions from the dplyr and tidyr packages.

To assess the relationships between the different variables, we performed a correlation analysis. This involved computing a pairwise Pearson correlation matrix using the cor() function on the selected numerical variables. The resulting matrix was then visualized as a correlogram using the corrplot() function from the corrplot package, which provided a clear graphical representation of the strength and direction of these relationships.

To model the relationships between our parameters and key rheological variables, we initially explored various machine learning regression models. Given the limited size of our dataset, we decided to focus on Support Vector Machine (SVM) regression, implemented with the svm() function from the e1071 package. SVM models are known to be robust and perform well even with smaller datasets. It is important to note that, due to the limited number of samples, we did not split the data into separate training and testing sets, and all models were trained on the full dataset. To assess the accuracy of our method and provide a fuller picture of predictive reliability, we used the coefficient of determination (R^2^), calculated between the real parameter values and those predicted by MIRANDA. A 95% confidence interval for the R^2^ (CI95%) values was also calculated using the Bias-Corrected and Accelerated (BCa) bootstrap method, as this approach is robust to skewness and bias in the bootstrap distribution.

Furthermore, to characterize the consistency and variability of our measurements across different replicates for each material, we computed several key descriptive statistics. We used functions from the dplyr package to calculate the mean, standard deviation (SD), number of replicates (N), and the coefficient of variation (CV) for each variable. The CV, expressed as a percentage, provides a standardized measure of dispersion, allowing for a direct comparison of variability between different variables. The Mean Absolute Deviation (MAD) was also calculated to provide a robust measure of variability, as it is less sensitive to outliers than the standard deviation. These statistics were then presented in a consolidated table to summarize the experimental results.

## 3. Results and Discussion

The results of our study are presented and discussed below, divided into three main sections: (1) variable correlation, (2) predictive modeling, and (3) system performance evaluation, in order to provide a comprehensive analysis of the data.

The experiment yielded 113 observations from 17 distinct material types. These included various SoftDoughs (plain, yellow, white), several foams (black, yellow, green, white), MaToni (human hand), Wood, Viscoelastic Material, Plasticine, a soft Toy (composed of wheat flour), Kinetic Sand, White Modeling Clay, and Stelan Flexible Paste. Each of these materials had either 3 or 6 general replicas. Additionally, five types of Flour doughs (with W-values of 90, 119, 262, 400, and 461) were tested. For these flour doughs, replicas were generated from a single prepared dough that was subsequently divided into two halves, with each half yielding its own set of replicas. A summary of all results can be found in Appendix B.

Table 1 provides a detailed breakdown of how each material tested is anticipated to behave from a viscoelastic perspective. This classification serves as a crucial reference for understanding the inherent properties and predicted responses of the samples under various conditions, offering a foundational context for the experimental results.

### 3.1. Graphical Correlation Analysis Between RELAPP and MIRANDA

A series of parameters and variables related to the viscoelasticity of materials have been calculated using the algorithms available in MIRANDA and RELAPP. The fitted mathematical models belong to the exponential family and have been chosen through trial and error:

RELAPP (Creep Phase):

- ffinal: final resistive force (Nw)
- a_exp: parameter ‘a’ of mathematical model m1
- b_exp: parameter ‘b’ of mathematical model m1
- c_exp: parameter ‘c’ of mathematical model m1
- a: parameter ‘a’ of mathematical model m2
- b: parameter ‘b’ of mathematical model m2

MIRANDA (Recovery Phase) (Everything is standardized to a scale of 0 to 100):

- Elasticity: geometric elasticity as a percentage; deformation recovery
- Final deformation: height of the maximum deformation
- Time of Recovery (TR): recovery time in video frame units
- Parameter a: parameter ‘a’ of mathematical model m2
- Parameter b: parameter ‘b’ of mathematical model m2

The parameters a and b for the 2-parameter exponential model, and a, b, and c for the 3-parameter exponential model, are explained in Appendix A, Section Mathematical Models and Creep-Recovery Curve Fitting. These models are based on exponential functions used in rheology to describe time-dependent deformation, and their parameters are calculated using a least-squares algorithm.

**Figure 5:**
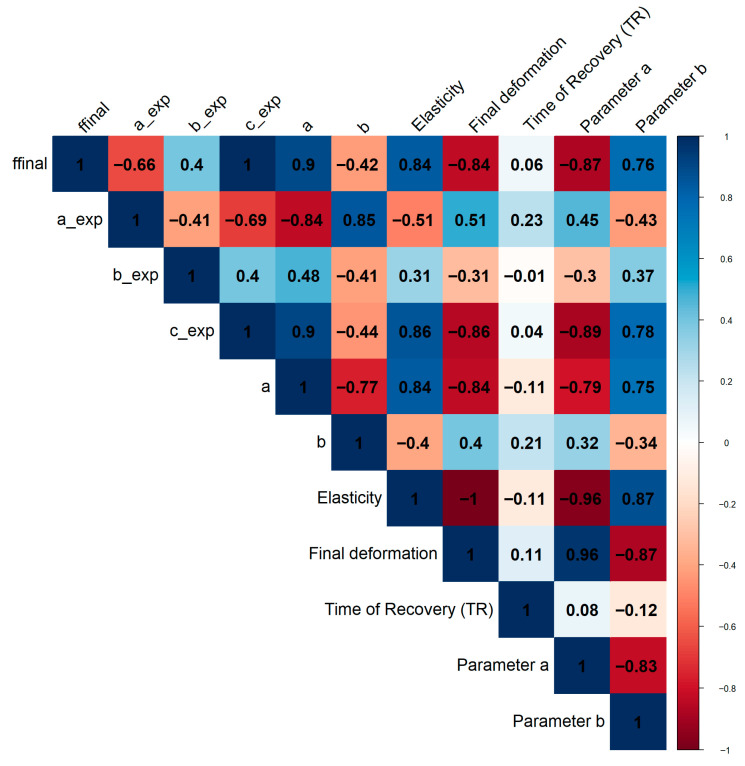
Correlation matrix between parameters and variables measured between MIRANDA and RELAPP (color intensity indicates more or less Pearson correlation, blue positive correlation, red negative correlation).

As shown in Figure 5, a Pearson correlation analysis was performed between the variables and parameters measured and estimated by MIRANDA and RELAPP, respectively. The most significant and interesting correlations were found between the creep phase parameters from RELAPP and the recovery phase parameters from MIRANDA.

The strongest positive correlations were observed between ffinal and Elasticity (0.839), and even more so between c_exp and Elasticity (0.858). This suggests that a higher final resistive force during the creep phase and a larger value for the c_exp parameter of model m1 are strongly linked to a greater geometric elasticity and deformation recovery during the recovery phase.

Conversely, strong negative correlations were found. For example, a high ffinal shows a strong inverse relationship with the Final deformation (−0.839), and a high c_exp shows a similar relationship with Final deformation (−0.858). This means that if a material offers more resistance during the creep phase (higher ffinal) and exhibits a larger c_exp value, it will have a smaller maximum deformation.

The strongest negative correlations were between ffinal and Parameter a (−0.872), and c_exp and Parameter a (−0.888). This indicates that the parameters defining the mathematical models are highly interconnected. A greater final resistive force or a higher c_exp value is associated with a smaller value for Parameter a from model m2.

#### Viscoelastic Interpretation

From a viscoelastic perspective, these correlations provide valuable insights into the material’s behavior. The strong positive correlation between ffinal/c_exp and Elasticity suggests that the energy stored during the creep phase (manifested as resistance) is efficiently released during the recovery phase. ffinal represents the material’s resistance to flow over time, while c_exp is a parameter from an exponential model likely related to the time constant of the viscoelastic response. A higher resistive force or a larger c_exp value implies a material with a more pronounced elastic component. This stored elastic energy drives the recovery process, leading to greater Elasticity or deformation recovery.

The equally strong negative correlation with Final deformation reinforces this interpretation. If a material is more resistant to deformation during the creep phase (high ffinal), it won’t deform as much in the first place, leading to a smaller final deformation. This highlights a fundamental trade-off: a material that resists deformation more effectively (stronger elastic component) will both deform less under load and recover more once the load is removed.

In essence, these results indicate that the two phases, creep and recovery, are intrinsically linked. The parameters calculated by RELAPP (creep) and MIRANDA (recovery) are not independent but are different manifestations of the same underlying viscoelastic properties. Materials that demonstrate a strong resistive or elastic-like behavior during the deformation process will subsequently exhibit a high degree of elastic recovery. While our model provides a robust empirical fit, future work could explore more complex analytical frameworks that link viscoelasticity to contact mechanics and material restitution [16].

### 3.2. Accuracy and Repeatability of the Method

This section evaluates the accuracy and repeatability of the MIRANDA method in characterizing the elasticity of various materials. Accuracy refers to how close the measured values are to the true values, while repeatability assesses the consistency of measurements under the same conditions. The analysis primarily focuses on the mean elasticity, its Coefficient of Variation (CV%), and Median Absolute Deviation (MAD) (See Table A1, Appendix B. Replica Analysis).

#### 3.2.1. Elasticity of Predominantly Elastic Materials

The MIRANDA method demonstrates strong performance in characterizing materials classified as 100% Elastic. For “Escuma negra” (Black Foam), a mean elasticity of 96.8% was observed. Crucially, this high mean was accompanied by a remarkably low Coefficient of Variation (CV%) of 0.915% and a Median Absolute Deviation (MAD) of 0.282. Similarly, “Escuma verda” (Green Foam) showed a mean elasticity of 88.650 with a CV% of 4.864% and MAD of 6.079. “Escuma blanca” (White Foam) also exhibited a high mean elasticity of 90.133 with a CV% of 9.034%. These results indicate that MIRANDA provides accurate and highly repeatable measurements for materials with a dominant elastic component.

**Figure 6:**
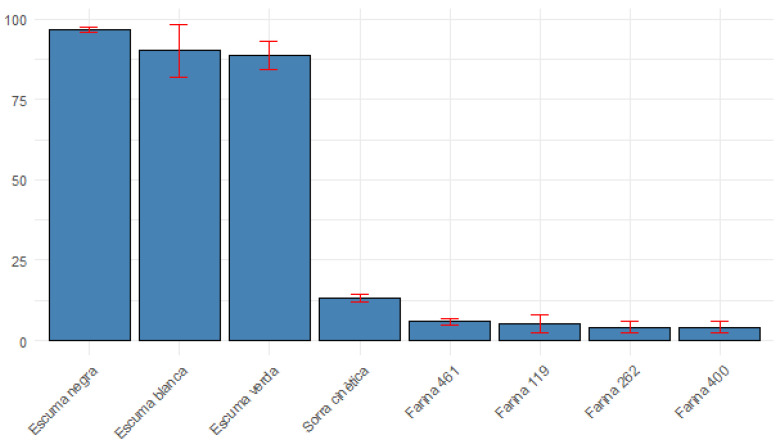
Elasticity of different materials and calculation of the coefficient of variation (CV%, red error bars).

In Figure 6, a graph presents different types of materials such as elastic foams, plastic material, and flour dough, showing the mean elasticity and its coefficient of variation (%).

#### 3.2.2. Elasticity of Plastic and Plasto-Visco-Elastic Materials

For “Sorra cinètica” (Kinetic Sand), categorized as highly plastic, the mean elasticity was 13.27%, with a CV% of 8.0% and MAD of 1.112. While lower than foams, the consistency of measurement for this material is still notable.

Materials classified as Plasto-Visco-Elastic, such as various flour doughs and soft doughs, presented a broader range of elasticity values and varying degrees of repeatability:

Flour doughs like “Farina 119”, “Farina 262”, and “Farina 400” showed relatively low mean elasticity values (5.200, 4.195, and 4.185, respectively), which is expected given their complex rheological behavior. However, these materials exhibited significantly higher CV% values for elasticity: 54.937% for “Farina 119”, 44.933% for “Farina 262”, and 44.775% for “Farina 400”.

For “Soft dough groga” (27.910), “Soft dough blanca” (13.080), “Pasta flexible Stelan” (6.330), and “Farina 90” (1.380), elasticity values were reported, but the CV% and MAD were marked as “NA” or “NaN” in the provided data, preventing an assessment of their repeatability.

A substantial portion of the dataset, including materials like “EscumaGroga”, “EscumaNegra”, “EscumaVerda” (from the MIRANDA section), “Fusta” (Wood), “Joguina” (soft Toy), “Pasta modelar blanca” (White Modeling Clay), “PlastilinaSemiPlastic” (Plasticine), “SoftDough”, “Viscoelastica”, and “maToni” (Human hand), lacked quantifiable elasticity, CV%, or MAD values (marked as “NaN” or “NA”). This absence of data limits a comprehensive evaluation of MIRANDA’s performance across these diverse material types.

The results strongly suggest that the MIRANDA method is highly effective in accurately and repeatably measuring the elastic properties of materials that are predominantly elastic, such as various foams. The consistently low CV% and MAD values for these materials underscore the method’s precision and reliability in such applications. The Coefficient of Variation (CV%) is a measure of relative variability, calculated as the standard deviation divided by the mean, expressed as a percentage (CV% = (SD/Mean) × 100). A lower CV% indicates higher precision and consistency in measurements, as observed with the foams. The Median Absolute Deviation (MAD) is a robust measure of statistical dispersion, less sensitive to outliers than the standard deviation, providing a more stable indication of data spread. The low MAD values for foams further confirm the tight clustering of their elasticity measurements.

However, the analysis also reveals challenges in the method’s repeatability for certain complex materials. The high CV% values observed for some flour doughs (“Farina 119”, “Farina 262”, “Farina 400”) indicate a significant degree of variability in the elasticity measurements for these materials. This could be attributed to several factors (independently of possible outliers):

- Inherent Material Variability: Plasto-visco-elastic materials like doughs can exhibit complex and highly sensitive rheological behaviors that are more susceptible to minor variations in sample preparation (such as kneading and handling of the sample) and environmental conditions like temperature and humidity.
- Method Sensitivity: While MIRANDA excels with highly elastic materials, its sensitivity to the subtle elastic components within predominantly viscous or plastic materials might lead to higher measurement noise.
- Limitations in Data Collection/Processing: The widespread presence of “NA” or “NaN” values for elasticity and its associated variability metrics for numerous materials is a critical limitation. This prevents a full assessment of the method’s performance across all material classifications and suggests either that elasticity was not a primary focus of measurement for these materials or that data acquisition/processing challenges were encountered.

The high CV% values observed in some samples, particularly in flour doughs, are directly influenced by the inherent variability of these plasto-visco-elastic substances. This variability is highly sensitive to subtle differences in sample preparation, such as kneading, as well as environmental factors like temperature and humidity. The robotic RELAPP system was specifically designed to minimize these procedural inconsistencies by providing a highly reproducible application of force, thereby attempting to mitigate the contribution of these factors to the measured variability.

The absence of complete data for all materials, particularly for the CV% and MAD for several plasto-visco-elastic substances, hinders a full understanding of MIRANDA’s repeatability across the entire spectrum of material types. Future investigations should prioritize comprehensive data collection for all relevant parameters to thoroughly assess the method’s robustness. Despite these limitations, the clear distinction in repeatability between elastic foams and more complex doughs provides valuable insights into the method’s strengths and areas requiring further refinement or specific calibration.

### 3.3. Application of MIRANDA in Predicting Dough Rheological and Industrial Characteristics

The Pearson correlation has been analyzed between rheological parameters measured in the lab and obtained by Mat Control laboratory from a proficiency test of measurement devices commonly found in flour mill laboratories, such as alveographs, amylographs, Brabender farinographs, and others [6].

The Alveograph measures a dough’s strength and extensibility by inflating a bubble, providing parameters like tenacity (P), extensibility (L), and baking strength (W); a high W indicates strong flour for bread, while low W suggests weaker flour for biscuits or cakes. The Amylograph assesses alpha-amylase activity in flour by measuring viscosity changes during heating, which helps determine enzyme activity for fermentation and crust formation. The Farinograph evaluates flour’s water absorption and mixing properties by measuring torque during mixing, yielding data on water absorption, dough development time, stability, and mixing tolerance. Five flour samples were taken and kneaded in a standardized manner using a laboratory mixer, forming dough with only salted water and flour. The main characteristics of these flours (alveograph values) are presented in Table 2, comprising two weak flours (W < 150), one medium flour (W < 300), and two strong flours (W > 300). In total, 14 samples of these flours were prepared and measured with MIRANDA and RELAPP.

**Figure 7:**
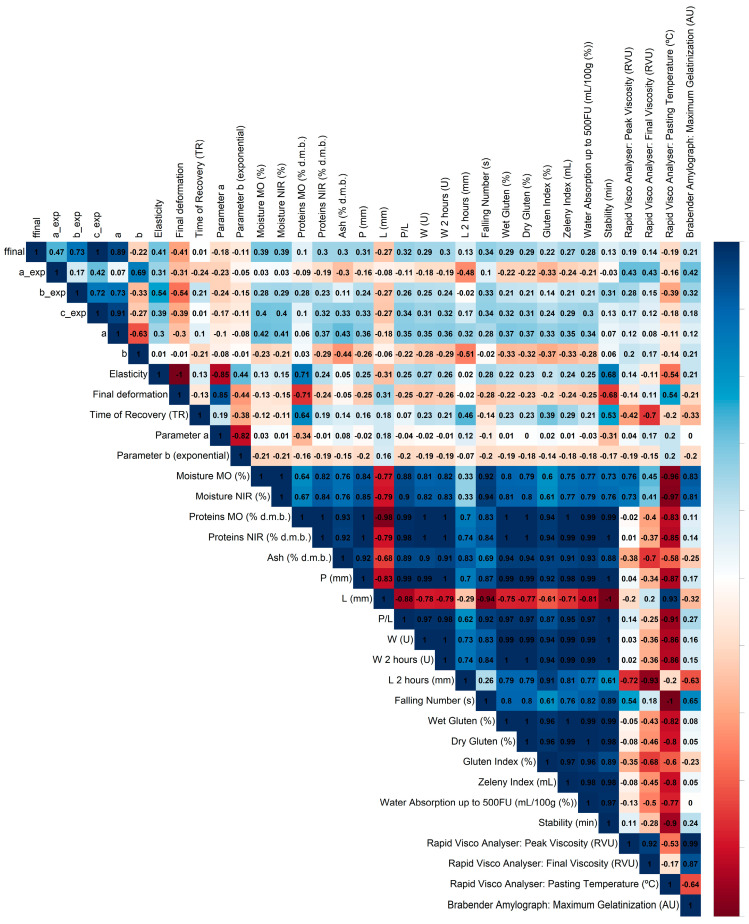
Pearson correlations between MIRANDA and RELAPP variables and reference values (calculated in a flour laboratory) used in the industrial process of flour quality control. The unit U for the variables W and W 2 h corresponds to 10^−4^ J.

This study specifically sought correlations between MIRANDA’s variables and the reference variables provided by MATCONTROL laboratory, which are presented in Figure 7.

Correlation analysis was performed between the MIRANDA system parameters and standard laboratory reference parameters for flour (Figure 7). By lowering the correlation threshold to |r| ≥ 0.6, several significant relationships were revealed that provide a more complete view of the MIRANDA system’s potential for flour characterization.

#### 3.3.1. Elasticity and Final Deformation as Indicators of Dough Strength

The Elasticity parameter exhibited a strong positive correlation with protein content (r = 0.715) and a positive correlation with Farinograph stability (r = 0.684). In contrast, the Final Deformation parameter was strongly and negatively correlated with protein content (r = −0.715) and negatively correlated with stability (r = −0.684).

The strong correlations of Elasticity and Final Deformation with both protein content and Farinograph stability consolidate their role as excellent indicators of dough strength. Farinograph stability measures a dough’s tolerance to intense mixing before its gluten structure begins to break down. It is logical that a flour with higher protein (r = 0.715) would form a stronger gluten network, resulting in higher Elasticity and greater mixing tolerance (stability, r = 0.684). Conversely, this same strong dough resists extension, which is reflected in its lower Final Deformation (r = −0.715).

From an industrial standpoint, these MIRANDA parameters are highly valuable for predicting dough behavior. A high Elasticity value suggests that the flour will produce robust doughs capable of withstanding long fermentation times and mechanical stress, making it ideal for industrial bread-making. A high Final Deformation value would signal a weak flour, resulting in a slack, difficult-to-handle dough with low processing tolerance, which would be more suitable for applications like cookies or certain pastries where a strong gluten network is not required.

#### 3.3.2. Time of Recovery (TR) as a Complex Indicator of Dough Structure

The Time of Recovery (TR) parameter showed a strong negative correlation with the Rapid Visco Analyser (RVA) final viscosity (r = −0.703) and a positive correlation with protein content (r = 0.642).

The Time of Recovery (TR) is revealed to be a more sophisticated parameter, influenced by both gluten and starch properties. The positive correlation with protein content (r = 0.642) suggests that stronger, more tenacious gluten networks require a longer time to recover their original shape after deformation. This is physically consistent, as a “tighter” gluten network would exert a powerful but slower recoil force.

Simultaneously, the negative correlation with RVA final viscosity (r = −0.703) is maintained. This indicates that a faster recovery time is associated with a starch structure that forms a more rigid gel upon cooling. Therefore, the TR parameter is uniquely positioned to differentiate between flours with similar protein content but different structural characteristics. A flour with a high TR would indicate a highly tenacious and elastic gluten, excellent for products requiring high gas retention (e.g., ciabatta, artisan breads). In contrast, a flour with a lower TR but high final viscosity might derive its favorable rheological behavior not just from gluten but also from its starch contribution, a key factor for influencing final product texture and shelf-life by managing moisture migration and staling.

#### 3.3.3. Internal Model Parameters

The mathematical model parameters, Parameter ‘a’ and Parameter ‘b’, did not show any significant correlation (|r| ≥ 0.6) with any of the reference flour parameters analyzed.

The lack of significant correlation for Parameter ‘a’ and Parameter ‘b’ with any standard quality metric, even at a less strict threshold, reinforces their conclusion as internal variables of the MIRANDA algorithm. For a quality control technician, these parameters do not serve as direct diagnostic indicators and cannot be used to certify that a flour meets a specific customer specification for protein, strength, or enzymatic activity. Their function appears to be purely computational within the device’s predictive model.

The results of this analysis highlight the capability of the MIRANDA system to predict key rheological properties of dough, with direct implications for industrial applications.

### 3.4. Predictive Models for Alveographic Parameters and Viscosity

This study aimed to develop predictive models for industrially relevant flour variables that did not exhibit a strong correlation with a single variable from MIRANDA measurements or existing reference values. Despite the limited sample size, the stable and easily interpretable variables TR and elasticity were utilized. A Support Vector Machine (SVM) regression model, implemented using the svm function from the e1071 R library, was employed for this purpose. The predictive performance was assessed using the coefficient of determination (R^2^) and statistical significance (*p*-value).

**Figure 8:**
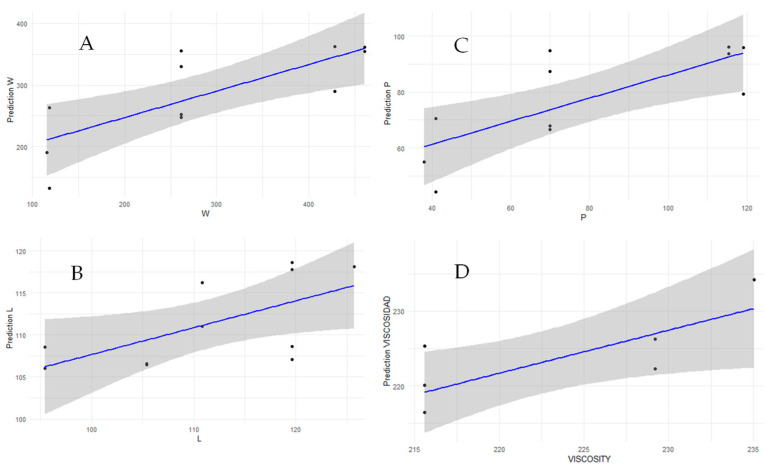
Regression plots between alveograph and viscosity values of flours, which were measured in the laboratory using standardized methods, and the values predicted by a machine learning model. This model predicts the same values using variables and parameters calculated by MIRANDA. The fit line (blue) and its 95% prediction interval are shown (where the points represent the analyzed samples and the gray shaded area is the prediction interval). (**A**) Measurement of W (baking strength), (**B**) L (alveograph extensibility), (**C**) P (tenacity), and (**D**) and Viscosity (Rapid Visco Analyser: Final Viscosity (RVU)). Units: W (10^−4^ J), L (mm), P (mm), Viscosity = Final Viscosity (RVU, 1 RVU = 12 cP).

For W (baking strength), a predictive R^2^ of 0.594 (CI95% [0.1191, 0.8558]) (Adjusted R^2^: 0.549, *p* = 0) was obtained when using elasticity and TR. For P (tenacity), the model yielded an R^2^ of 0.575 (CI95% [0.0862, 0.8470]) (Adjusted R^2^: 0.5277, *p* = 0). The prediction for L (extensibility) showed an R^2^ of 0.3944 (CI95% [0.0096, 0.8022]) (Adjusted R^2^: 0.327, *p*-value: 0.03856). Finally, for viscosity measured as Final Viscosity (RVU) by a Rapid Visco Analyser, a predictive R^2^ of 0.612 (CI95% [0.2100, 0.7828]) (Adjusted R^2^: 0.534, *p*-value: 0.03763) was achieved. These regression plots, illustrating the fit line and 95% prediction interval between observed alveographic and viscosity values and those predicted by the MIRANDA-based machine learning model, are presented in Figure 8.

Despite the admittedly small sample size, the preliminary results demonstrate the potential of MIRANDA variables (TR and elasticity) in conjunction with machine learning models to predict key alveographic parameters (W, P, L) and viscosity (Final Viscosity (RVU) using a Rapid Visco Analyser). The statistically significant *p*-values, particularly for W, P, and final viscosity, indicate that the observed correlations are unlikely due to random chance, even with fewer data points. The R^2^ values, ranging from approximately 0.39 to 0.61, suggest a moderate to good predictive capability for these models.

This has significant implications for industrial quality control:

- Accelerated Development Cycles: The ability to predict traditional rheological parameters quickly using MIRANDA could drastically reduce the time needed for R&D and new product development in flour milling and baking industries.
- Enhanced Continuous Quality Control: Rapid, non-destructive measurements from MIRANDA could enable real-time monitoring of flour quality, allowing for immediate adjustments in production and minimizing off-spec products.
- Reduced Reliance on Traditional Methods: While traditional methods are crucial for reference, the predictive models could offer a faster, more cost-effective alternative for routine checks, especially for parameters that are time-consuming to measure with conventional equipment.
- Insight into Material Behavior: The correlations, even if not perfect, provide valuable insights into how MIRANDA ‘s measured variables relate to established rheological properties, deepening our understanding of dough mechanics.

While these findings are promising, it is crucial to acknowledge that the limited sample size necessitates further validation with a larger, more diverse dataset to confirm the robustness and generalizability of these predictive models. However, the initial success strongly advocates for continued research and development in integrating novel sensing technologies like MIRANDA into industrial quality control workflows.

While these findings are promising, we acknowledge that the limited sample size of 113 observations represents a key limitation. This size may lead to models that are overfitted to our specific dataset, potentially hindering their performance and generalizability to a wider range of flour varieties, compositions, and origins. We therefore consider these results a proof of concept that strongly advocates for future validation with a larger, more diverse dataset to confirm the robustness and broader applicability of these predictive models for industrial workflows.

### 3.5. Comparison with Traditional Rheometers

To validate the accuracy of our dual MIRANDA-RELAPP system, we directly compared its outputs with established, standardized rheological methods. This validation is demonstrated quantitatively by the predictive models presented in the previous section (Section 3.4) and is visually supported by the regression plots in Figure 8. The strong and statistically significant correlations (as evidenced by the R^2^ and *p*-values) obtained between our models’ predictions and the values measured by a Chopin Alveograph and a Rapid Visco Analyser serve as the primary evidence of our system’s accuracy.

Specifically, the ability of MIRANDA’s TR and elasticity variables to predict key alveographic parameters such as baking strength (W, R^2^ = 0.594 (CI95% [0.1191, 0.8558]) and tenacity (P, R^2^ = 0.575 CI95% [0.0862, 0.8470]) validates that our rapid, non-destructive approach can accurately characterize fundamental material properties. This confirms the potential of our system to serve as a reliable, cost-effective, and faster alternative for quality control and research, complementing and, in some cases, substituting the more time-consuming and expensive traditional rheometers.

## 4. Conclusions

Our results confirm that MIRANDA’s computer vision and advanced algorithms reliably predict complex rheological properties that traditionally require direct contact and active deformation. Strong correlations were consistently observed between the variables of both systems, particularly excellent correlations between MIRANDA’s Elasticity and RELAPP’s c_exp (r = 0.858) and final resistive force (r = 0.839), confirming the synergy between the systems. Furthermore, our predictive models for industrially relevant parameters like baking strength (W), tenacity (P), and especially with viscosity (RVU) show promising results.

These findings have significant implications for industrial applications. The speed, portability, and cost-effectiveness of our system offer a clear pathway for its integration into production lines, enabling real-time and continuous quality control. This can accelerate product development cycles and reduce reliance on slower, more expensive traditional methods.

Finally, while the results are highly promising, future work will focus on enhancing the model’s robustness and generalizability. This involves not only validation with larger and more diverse datasets but also refining our approach by incorporating a more comprehensive set of material properties, such as viscosity and rate-dependent behavior. These next steps will be crucial for confirming the broad applicability and accuracy of our system.

## 5. Patents

The patentability of the RELAPP robotic system is currently under evaluation by the participating institutions. The MIRANDA software is registered by the University of Barcelona in accordance with the intellectual property laws that regulate such systems. Additionally, the complementary software RELAPP Analyzer, a Shiny-based application developed for the analysis of data obtained from RELAPP’s sensors, is currently in the process of registration.

## Figures and Tables

**Table 1:** Expert classification of the materials based on their expected viscoelastic behavior, as indicated.

Matherial Type	Description
Escuma Negra (Black Foam), Escuma Groga (Yellow Foam), Escuma Verda (Green Foam), Escuma Blanca (White Foam)	100% Elastic (They exhibit purely elastic behavior, deforming under stress and returning to their original shape when the stress is removed without energy dissipation).
Fusta (Wood)	Rigid (It is a stiff material that resists deformation under applied force, with minimal change in shape or volume).
Sorra Cinètica (Kinetic Sand) All Other Materials	100% Plastic (It behaves as a purely plastic material, deforming permanently once a certain stress threshold is exceeded, without recovering its original shape). Plasto-Visco-Elastic (These materials exhibit a combination of plastic, viscous, and elastic behaviors. They can deform elastically, flow viscously over time, and also undergo permanent deformation (plasticity) when sufficiently stressed.

**Table 2:** Main alveograph characteristics of the flour doughs used and analyzed by RELAPP, MIRANDA, and their reference alveograph values (W = baking strength, P = tenacity, L = extensibility).

Material (Label)	P (mm)	L (mm)	W (10^−4^ J)
Farina 90	38.091	125.693	115.796
Farina 119	40.913	110.834	118.500
Farina 262	69.894	119.646	261.522
Farina 400	119.039	95.471	428.033
Farina 461	115.334	105.414	460.580

**Figure A1:**
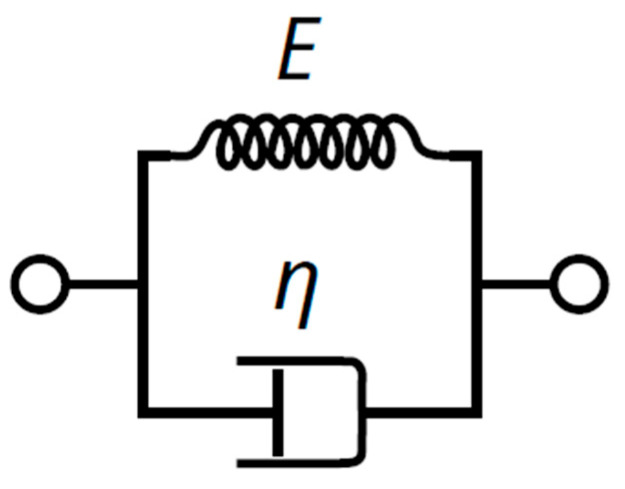
Spring-and-damper model upon which the Kelvin-Voigt model is based. It accurately describes creep, where deformation increases over time under a constant stress.

**Figure A2:**
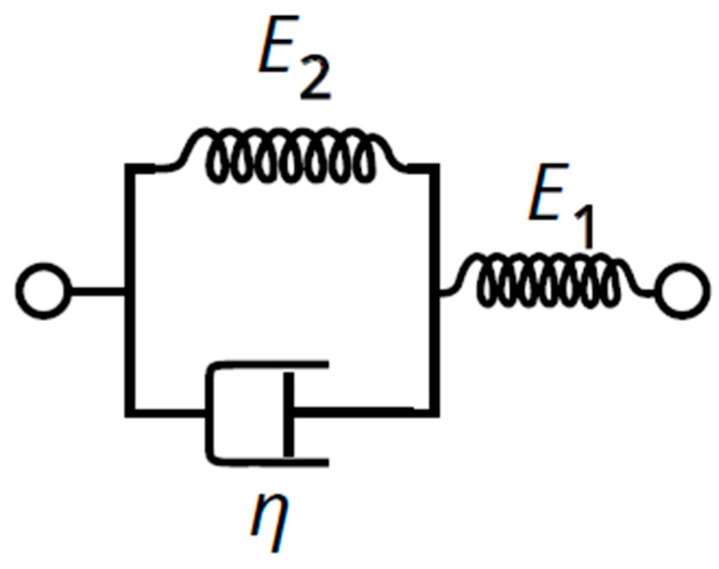
Spring-and-damper model upon which the SLT model is based: More realistic, it combines instantaneous deformation with delayed deformation.

## Data Availability

All aggregated results generated during this study are included in Table A1 of Annex B, allowing for the verification of the presented statistical analyses. The raw data have not been included as they require several pre-processing steps before analysis, but are available from the corresponding author upon reasonable request.

## Appendix A

The behavior of deformable materials is defined by the relationship between stress and strain [13]. Solids can be elastic, with an instantaneous spring-like response, or viscoelastic, showing a time-dependent behavior like doughs. This unique viscoelastic “fingerprint” is characterized using the Creep-Recovery Test, which is the method employed by your MIRANDA system.

### Appendix A.1. Fundamental Concepts of Deformation

The state of a deformable solid can be described using fundamental fields:

- Displacement Field ($\vec{u}\left(\vec{x},t\right)$): This field describes the position of each point within the material over time.
- Strain Tensor ($\epsilon \left(\vec{x},t\right))$: Represents how the material locally changes shape (stretching and angular distortion). For small deformations, the strain tensor is given by:
  $$\epsilon =\frac{1}{2}[\nabla \vec{u}+{\left(\nabla \vec{u}\right)}^{T}]$$
- Stress Tensor ($\sigma \left(\vec{x},t\right))$: This tensor represents the internal forces acting within the material, describing the distribution of forces per unit area.

The fundamental question in material characterization is to establish the relationship between stress (σ) and strain (ϵ), which is defined by the material’s constitutive equation.

#### Appendix A.1.1. Constitutive Equations: Elastic vs. Viscoelastic Behavior

The constitutive equation is the “fingerprint” of a material, defining its behavior when deformed under stress.

- Elastic Solid:
  ○ Exhibits instantaneous and reversible response to applied stress.
  ○ Stores energy, similar to a spring.
  ○ Can be modeled by Hooke’s Law: σ=C/ϵ, where C is a stiffness tensor.
  ○ Upon removal of the force, the material instantly returns to its original shape.
- Viscoelastic Solid:
  ○ Exhibits time-dependent response.
  ○ Both stores and dissipates energy, behaving like a combination of a spring and a damper.
  ○ The stress depends on the entire history of the deformation:

$$\sigma \left(t\right)=F[\epsilon \left(t^{\prime }\right),\;t^{\prime }\leq t]$$

#### Appendix A.1.2. Rheological Models for Viscoelasticity

For greater accuracy, generalized models (like the Wiechert model) use multiple spring-damper elements to represent a spectrum of relaxation times, better describing complex materials [17]. The basis for viscous behavior is derived from principles like those described by the Stokes equations, which establish the drag force in a fluid and underpin the response of the damper [18]. The Kelvin-Voigt and Standard Linear Solid (SLS) models are foundational frameworks for predicting how viscoelastic materials behave by combining simple spring (elastic) and damper (viscous) elements. The Kelvin-Voigt model excels at describing creep, while the more realistic SLS model captures both creep and stress relaxation. Their importance lies in enabling the design and characterization of materials like polymers and tissues, though more advanced models exist for greater accuracy.

To model viscoelastic behavior, simple mechanical elements (springs for elastic response and damper s for viscous response) are combined:

- Kelvin-Voigt Model:
  ○ Composed of a spring and a damper in parallel.
  ○ Its constitutive equation is: $\sigma =\;E\epsilon +\;\eta \dot{\epsilon }$, where E is the elastic modulus and η is the viscosity.
  ○ This model accurately describes creep, where the deformation increases over time under constant stress.

- Standard Linear Solid (SLS) Model:
  ○ More realistic, combining instantaneous and retarded deformation.
  ○ Typically represented by a Kelvin-Voigt element in series with a spring.
  ○ Its constitutive equation is: $\sigma +\tau_{\epsilon }\dot{\sigma }=E_{R}(\epsilon +\;\tau_{\sigma }\dot{\epsilon })$, where $\tau_{\epsilon }$ and $\tau_{\sigma }$ are relaxation times and $E_{R}$ is the relaxed modulus.

#### Appendix A.1.3. Creep-Recovery Test

The Creep-Recovery Test is a common experimental procedure used to characterize viscoelastic materials, which is the type of experiment analyzed by MIRANDA and RELAPP. It consists of two phases:

- Creep Phase: This phase, adapted for our robotic system, involves the robot’s arm engaging the material by pushing it to a pre-defined target position. While the robot aims to maintain this set position, its inherent flexibility, coupled with the material’s viscoelastic properties (like doughs), results in a progressive, coupled deformation. During this unique interaction, the evolving force exerted by the material back on the robot is continuously measured, providing a characteristic “fingerprint” of the material’s viscoelastic response under these specific, controlled conditions.
- Recovery Phase: The applied stress is removed. The material then recovers (or partially recovers) its shape, usually in a delayed and often incomplete manner.

The shape of the displacement curve u(t) (or a signal proportional to it) obtained from a Creep-Recovery Test serves as a unique “fingerprint” for each material. While ideal models often describe material behavior in terms of deformation ϵ(t), for our systems, a reasonable hypothesis is that the measured displacement is proportional to the material’s deformation, such that ϵ(t)∝u(t). The objective of our developed systems, MIRANDA and RELAPP, is to capture and quantify this displacement “fingerprint” using computer vision, robotics, and deep learning techniques.

##### Mathematical Models and Creep-Recovery Curve Fitting

The mathematical models for the creep-recovery analysis are based on widely accepted exponential-family functions used in the rheology of viscoelastic materials such as doughs. These models are chosen for their proven ability to accurately describe time-dependent deformation. The proposed two-parameter model for the creep phase is given by $y\left(t\right)=C{\left(t+1\right)}^{a}$, which can be rewritten as a two parameters model (model 2: m2) $y\left(t\right)=e^{\left(a\log\left(t+1\right)+b\right)}$, where ${C=e}^{b}$**.** From a physical perspective, the parameter *a* act as a power-law exponent that governs the rate of displacement over time. When 0 < *a* < 1, the model describes a decelerating movement, consistent with the relaxation behavior of a viscoelastic material. The parameter *b* (or *C*) is a scaling factor that represents the magnitude of the displacement for a given time and is related to the initial conditions of the system. A three-parameter exponential function (model 1: m1) of the form $y\left(t\right)={Ae}^{\left(Bt\right)}+C,$ was also initially considered, but was ultimately discarded. The parameters for both models were determined through an empirical non-linear regression process. The primary objective of this fitting was to obtain the most representative parameters directly from the experimental data, which is standard practice in experimental rheology. The fitting was performed using a least-squares algorithm to minimize the sum of squared differences between the observed data from MIRANDA and the values predicted by the models. Preliminary model fitting and validation were carried out using the nls() function in the R statistical programming environment. The two-parameter exponential model was ultimately chosen for the final analysis due to its superior numerical stability and its generally higher pseudo R-squared values across all material types. The three-parameter model, in contrast, was discarded because it frequently encountered numerical errors during parameter estimation and yielded lower R-squared values, making it a less reliable fit for the observed data. This robust methodology was subsequently adapted to Python for its integration into the MIRANDA software, ensuring that all calculations are both precise and reproducible. To assess the quality of the fit for each curve, a pseudo R-squared value was calculated, providing a measure of how well the model accounts for the observed variability in the data. This rigorous fitting process ensures that the chosen parameters accurately reflect the unique rheological fingerprint of each material sample tested.

## Appendix B

The results obtained in the experiment includes 113 observations (experiments) from 17 distinct types of materials tested:

- SoftDough (Soft Dough)
- Escuma Negra (Black Foam)
- Escuma Groga (Yellow Foam)
- MaToni (Human hand)
- Fusta (Wood)
- Viscoelastica (Viscoelastic Material)
- Plastilina SemiPlastic (Plasticine)
- Joguina (soft Toy composed by wheat flour)
- Escuma Verda (Green Foam)
- Escuma Blanca (White Foam)
- Soft dough groga (Yellow Soft Dough)
- Farina (Flour dought—followed by W-value, dough preparation, and half division)
  ○ Farina 90 (Flour W = 90)
  ○ Farina 262 (Flour W = 262)
  ○ Farina 400 (Flour W = 400)
  ○ Farina 461 (Flour W = 461)
  ○ Farina 119 (Flour W = 119)
- Sorra Cinètica (Kinetic Sand)
- Pasta Modelar Blanca (White Modeling Clay)
- Soft dough blanca (White Soft Dough)
- Pasta Flexible Stelan (Stelan Flexible Paste)

### Replica Analysis

Below is a breakdown of the number of replicas for each material type, distinguishing between general replicas and those derived from a single divided dough.

Materials with General Replicas (Not Divided Doughs):

- SoftDough: 6 replicas
- Escuma Negra (Black Foam): 6 replicas (3 EscumaNegra, 3 Escuma negra)
- Escuma Groga (Yellow Foam): 3 replicas
- MaToni: 3 replicas
- Fusta (Wood): 3 replicas (Fusta appears 3 times, with two entries separated, implying 3 distinct samples)
- Viscoelastica (Viscoelastic Material): 6 replicas
- Plastilina SemiPlastic (Plasticine): 3 replicas
- Joguina (Toy): 6 replicas
- Escuma Verda (Green Foam): 6 replicas (3 EscumaVerda, 3 Escuma verda)
- Escuma Blanca (White Foam): 6 replicas
- Soft dough groga (Yellow Soft Dough): 3 replicas
- Sorra Cinètica (Kinetic Sand): 3 replicas
- Pasta Modelar Blanca (White Modeling Clay): 6 replicas
- Soft dough blanca (White Soft Dough): 3 replicas
- Pasta Flexible Stelan (Stelan Flexible Paste): 3 replicas

Flour Materials: Replicas from Divided Doughs: For the ‘Farina’ (Flour) materials, the columns ‘pastada’ and ‘meitat’ indicate that a single dough was prepared (‘pastada’) and then divided into two halves (‘meitat’). Each ‘meitat’ then has its own replicas.

**Table A1:** Showing the statistics (Mitjana (mean), SD, N, CV (%) and MAD) of the different materials tested with RELAPP and MIRANDA. NaN (Not a Number) indicates missing data, meaning the value was unavailable or could not be computed.

		RELAPP						MIRANDA					
Material	Statistics	Ffinal	a_exp	b_exp	c_exp	a	b	Elasticity	Maximum Deformation	Final Deformation	Time of Recovery (TR)	Parameter a (Exponential)	Parameter b (Exponential)
Escuma blanca	Mitjana	18.953	3.960	−0.001	18.500	−0.070	3.475	90.133	0.997	0.099	1.847	−0.004	0.052
Escuma blanca	SD	0.103	0.169	0.000	0.005	0.001	0.002	8.142	0.001	0.081	0.695	0.001	0.000
Escuma blanca	N	3.000	3.000	3.000	3.000	3.000	3.000	3.000	3.000	3.000	3.000	3.000	3.000
Escuma blanca	CV (%)	0.543	4.269	−6.195	0.026	−0.969	0.052	9.034	0.116	82.524	37.651	−12.191	0.686
Escuma blanca	MAD	0.090	0.170	0.000	0.001	0.001	0.002	4.937	0.001	0.049	0.979	0.001	0.000
Escuma negra	Mitjana	21.990	3.546	−0.001	21.552	−0.049	3.466	96.747	0.999	0.033	2.287	−0.006	0.049
Escuma negra	SD	0.371	0.956	0.000	0.477	0.009	0.068	0.886	0.001	0.009	0.354	0.000	0.001
Escuma negra	N	6.000	6.000	6.000	6.000	6.000	6.000	3.000	3.000	3.000	3.000	3.000	3.000
Escuma negra	CV (%)	1.687	26.955	−10.789	2.214	−18.446	1.958	0.915	0.080	27.220	15.476	−4.660	2.987
Escuma negra	MAD	0.398	0.796	0.000	0.599	0.010	0.065	0.282	0.001	0.003	0.252	0.000	0.000
Escuma verda	Mitjana	18.939	4.473	−0.001	18.438	−0.078	3.530	88.650	0.997	0.114	1.300	−0.004	0.051
Escuma verda	SD	NA	NA	NA	NA	NA	NA	4.312	0.002	0.043	0.596	0.000	0.002
Escuma verda	N	1.000	1.000	1.000	1.000	1.000	1.000	3.000	3.000	3.000	3.000	3.000	3.000
Escuma verda	CV (%)	NA	NA	NA	NA	NA	NA	4.864	0.241	37.989	45.826	−7.830	3.839
Escuma verda	MAD	0.000	0.000	0.000	0.000	0.000	0.000	6.079	0.000	0.061	0.267	0.000	0.002
EscumaGroga	Mitjana	21.637	3.103	−0.001	21.203	−0.048	3.441	NaN	NaN	NaN	NaN	NaN	NaN
EscumaGroga	SD	NA	NA	NA	NA	NA	NA	NA	NA	NA	NA	NA	NA
EscumaGroga	N	1.000	1.000	1.000	1.000	1.000	1.000	0.000	0.000	0.000	0.000	0.000	0.000
EscumaGroga	CV (%)	NA	NA	NA	NA	NA	NA	NA	NA	NA	NA	NA	NA
EscumaGroga	MAD	0.000	0.000	0.000	0.000	0.000	0.000	NA	NA	NA	NA	NA	NA
EscumaNegra	Mitjana	24.278	3.320	−0.001	23.789	−0.042	3.514	NaN	NaN	NaN	NaN	NaN	NaN
EscumaNegra	SD	NA	NA	NA	NA	NA	NA	NA	NA	NA	NA	NA	NA
EscumaNegra	N	1.000	1.000	1.000	1.000	1.000	1.000	0.000	0.000	0.000	0.000	0.000	0.000
EscumaNegra	CV (%)	NA	NA	NA	NA	NA	NA	NA	NA	NA	NA	NA	NA
EscumaNegra	MAD	0.000	0.000	0.000	0.000	0.000	0.000	NA	NA	NA	NA	NA	NA
EscumaVerda	Mitjana	19.407	5.289	−0.001	18.849	−0.087	3.630	NaN	NaN	NaN	NaN	NaN	NaN
EscumaVerda	SD	0.564	0.706	0.000	0.774	0.010	0.050	NA	NA	NA	NA	NA	NA
EscumaVerda	N	2.000	2.000	2.000	2.000	2.000	2.000	0.000	0.000	0.000	0.000	0.000	0.000
EscumaVerda	CV (%)	2.907	13.341	−7.526	4.108	−11.735	1.390	NA	NA	NA	NA	NA	NA
EscumaVerda	MAD	0.591	0.740	0.000	0.812	0.011	0.053	NA	NA	NA	NA	NA	NA
Farina 119	Mitjana	7.669	7.935	−0.001	6.988	−0.285	4.204	5.200	0.995	0.948	1.785	0.000	0.046
Farina 119	SD	0.416	0.585	0.000	0.377	0.005	0.024	2.857	0.005	0.029	1.082	0.000	0.000
Farina 119	N	2.000	2.000	2.000	2.000	2.000	2.000	2.000	2.000	2.000	2.000	2.000	2.000
Farina 119	CV (%)	5.431	7.367	−0.701	5.397	−1.614	0.578	54.937	0.490	3.013	60.609	−124.512	0.663
Farina 119	MAD	0.437	0.613	0.000	0.395	0.005	0.025	2.995	0.005	0.030	1.134	0.000	0.000
Farina 262	Mitjana	7.820	6.864	−0.001	7.243	−0.265	4.046	4.195	0.999	0.958	2.545	0.000	0.046
Farina 262	SD	1.380	0.666	0.000	1.312	0.031	0.101	1.885	0.001	0.019	0.047	0.000	0.000
Farina 262	N	4.000	4.000	4.000	4.000	4.000	4.000	4.000	4.000	4.000	4.000	4.000	4.000
Farina 262	CV (%)	17.643	9.707	−3.700	18.115	−11.857	2.493	44.933	0.096	1.967	1.829	−122.866	0.315
Farina 262	MAD	1.207	0.688	0.000	1.094	0.014	0.104	1.520	0.001	0.015	0.030	0.000	0.000
Farina 400	Mitjana	8.775	7.278	−0.001	8.153	−0.247	4.043	4.185	0.999	0.958	1.730	0.000	0.046
Farina 400	SD	0.081	0.159	0.000	0.085	0.005	0.043	1.874	0.001	0.019	1.103	0.000	0.000
Farina 400	N	2.000	2.000	2.000	2.000	2.000	2.000	2.000	2.000	2.000	2.000	2.000	2.000
Farina 400	CV (%)	0.922	2.180	−1.407	1.039	−1.937	1.066	44.775	0.057	1.956	63.762	−28.284	0.295
Farina 400	MAD	0.085	0.166	0.000	0.089	0.005	0.045	1.964	0.001	0.020	1.156	0.000	0.000
Farina 461	Mitjana	8.428	7.465	−0.001	7.764	−0.258	4.081	5.915	0.991	0.941	2.490	0.000	0.046
Farina 461	SD	1.119	0.217	0.000	1.053	0.024	0.047	0.983	0.011	0.010	0.028	0.000	0.000
Farina 461	N	2.000	2.000	2.000	2.000	2.000	2.000	2.000	2.000	2.000	2.000	2.000	2.000
Farina 461	CV (%)	13.272	2.910	−3.050	13.568	−9.337	1.146	16.617	1.149	1.045	1.136	−150.192	0.850
Farina 461	MAD	1.173	0.228	0.000	1.104	0.025	0.049	1.030	0.012	0.010	0.030	0.000	0.000
Farina 90	Mitjana	8.055	7.491	−0.001	7.425	−0.262	4.069	1.380	0.991	0.986	1.410	0.000	0.046
Farina 90	SD	0.932	0.918	0.000	0.853	0.010	0.060	NA	NA	NA	NA	NA	NA
Farina 90	N	4.000	4.000	4.000	4.000	4.000	4.000	1.000	1.000	1.000	1.000	1.000	1.000
Farina 90	CV (%)	11.567	12.249	−1.257	11.495	−3.868	1.467	NA	NA	NA	NA	NA	NA
Farina 90	MAD	0.954	0.673	0.000	0.923	0.010	0.020	0.000	0.000	0.000	0.000	0.000	0.000
Fusta	Mitjana	29.187	0.924	−0.001	28.473	−0.006	3.421	NaN	NaN	NaN	NaN	NaN	NaN
Fusta	SD	0.367	1.472	0.001	1.420	0.005	0.034	NA	NA	NA	NA	NA	NA
Fusta	N	3.000	3.000	3.000	3.000	3.000	3.000	0.000	0.000	0.000	0.000	0.000	0.000
Fusta	CV (%)	1.257	159.338	−87.269	4.987	−75.513	0.982	NA	NA	NA	NA	NA	NA
Fusta	MAD	0.317	0.023	0.001	1.033	0.002	0.018	NA	NA	NA	NA	NA	NA
Joguina	Mitjana	22.661	0.234	−0.001	22.704	−0.012	3.208	NaN	NaN	NaN	NaN	NaN	NaN
Joguina	SD	0.408	0.287	0.001	0.346	0.008	0.046	NA	NA	NA	NA	NA	NA
Joguina	N	2.000	2.000	2.000	2.000	2.000	2.000	0.000	0.000	0.000	0.000	0.000	0.000
Joguina	CV (%)	1.801	122.982	−93.260	1.524	−70.094	1.422	NA	NA	NA	NA	NA	NA
Joguina	MAD	0.428	0.301	0.001	0.363	0.009	0.048	NA	NA	NA	NA	NA	NA
Pasta flexible Stelan	Mitjana	8.547	13.316	−0.001	7.414	−0.308	4.543	6.330	0.995	0.937	2.520	0.000	0.046
Pasta flexible Stelan	SD	NA	NA	NA	NA	NA	NA	NA	NA	NA	NA	NA	NA
Pasta flexible Stelan	N	1.000	1.000	1.000	1.000	1.000	1.000	1.000	1.000	1.000	1.000	1.000	1.000
Pasta flexible Stelan	CV (%)	NA	NA	NA	NA	NA	NA	NA	NA	NA	NA	NA	NA
Pasta flexible Stelan	MAD	0.000	0.000	0.000	0.000	0.000	0.000	0.000	0.000	0.000	0.000	0.000	0.000
Pasta modelar blanca	Mitjana	23.891	4.444	−0.001	23.095	−0.055	3.599	NaN	NaN	NaN	NaN	NaN	NaN
Pasta modelar blanca	SD	0.581	0.300	0.000	0.664	0.000	0.024	NA	NA	NA	NA	NA	NA
Pasta modelar blanca	N	2.000	2.000	2.000	2.000	2.000	2.000	0.000	0.000	0.000	0.000	0.000	0.000
Pasta modelar blanca	CV (%)	2.431	6.745	−8.772	2.876	−0.528	0.662	NA	NA	NA	NA	NA	NA
Pasta modelar blanca	MAD	0.609	0.314	0.000	0.696	0.000	0.025	NA	NA	NA	NA	NA	NA
PlastilinaSemiPlastic	Mitjana	6.135	10.062	−0.002	6.279	−0.346	4.441	NaN	NaN	NaN	NaN	NaN	NaN
PlastilinaSemiPlastic	SD	NA	NA	NA	NA	NA	NA	NA	NA	NA	NA	NA	NA
PlastilinaSemiPlastic	N	1.000	1.000	1.000	1.000	1.000	1.000	0.000	0.000	0.000	0.000	0.000	0.000
PlastilinaSemiPlastic	CV (%)	NA	NA	NA	NA	NA	NA	NA	NA	NA	NA	NA	NA
PlastilinaSemiPlastic	MAD	0.000	0.000	0.000	0.000	0.000	0.000	NA	NA	NA	NA	NA	NA
Soft dough blanca	Mitjana	6.638	6.448	−0.001	6.512	−0.308	4.226	13.080	1.000	0.869	2.330	0.000	0.046
Soft dough blanca	SD	NA	NA	NA	NA	NA	NA	NA	NA	NA	NA	NA	NA
Soft dough blanca	N	1.000	1.000	1.000	1.000	1.000	1.000	1.000	1.000	1.000	1.000	1.000	1.000
Soft dough blanca	CV (%)	NA	NA	NA	NA	NA	NA	NA	NA	NA	NA	NA	NA
Soft dough blanca	MAD	0.000	0.000	0.000	0.000	0.000	0.000	0.000	0.000	0.000	0.000	0.000	0.000
Soft dough groga	Mitjana	9.306	7.320	−0.001	8.476	−0.226	3.956	27.910	0.999	0.721	2.780	−0.001	0.046
Soft dough groga	SD	NA	NA	NA	NA	NA	NA	NA	NA	NA	NA	NA	NA
Soft dough groga	N	1.000	1.000	1.000	1.000	1.000	1.000	1.000	1.000	1.000	1.000	1.000	1.000
Soft dough groga	CV (%)	NA	NA	NA	NA	NA	NA	NA	NA	NA	NA	NA	NA
Soft dough groga	MAD	0.000	0.000	0.000	0.000	0.000	0.000	0.000	0.000	0.000	0.000	0.000	0.000
SoftDough	Mitjana	9.097	9.431	−0.001	8.346	−0.269	4.256	NaN	NaN	NaN	NaN	NaN	NaN
SoftDough	SD	1.334	0.245	0.000	1.251	0.025	0.036	NA	NA	NA	NA	NA	NA
SoftDough	N	2.000	2.000	2.000	2.000	2.000	2.000	0.000	0.000	0.000	0.000	0.000	0.000
SoftDough	CV (%)	14.668	2.599	−5.424	14.992	−9.211	0.850	NA	NA	NA	NA	NA	NA
SoftDough	MAD	1.399	0.257	0.000	1.312	0.026	0.038	NA	NA	NA	NA	NA	NA
Sorra cinètica	Mitjana	5.902	8.503	−0.001	5.219	−0.358	4.498	13.270	0.999	0.867	2.060	0.000	0.046
Sorra cinètica	SD	0.775	0.861	0.000	0.706	0.023	0.063	1.061	0.000	0.011	0.184	0.000	0.000
Sorra cinètica	N	3.000	3.000	3.000	3.000	3.000	3.000	2.000	2.000	2.000	2.000	2.000	2.000
Sorra cinètica	CV (%)	13.126	10.125	−0.482	13.523	−6.479	1.407	7.993	0.035	1.223	8.925	−44.698	0.063
Sorra cinètica	MAD	0.041	1.182	0.000	0.137	0.022	0.022	1.112	0.000	0.011	0.193	0.000	0.000
Viscoelastica	Mitjana	19.525	3.953	−0.001	18.995	−0.074	3.529	NaN	NaN	NaN	NaN	NaN	NaN
Viscoelastica	SD	0.804	0.614	0.000	0.723	0.007	0.012	NA	NA	NA	NA	NA	NA
Viscoelastica	N	2.000	2.000	2.000	2.000	2.000	2.000	0.000	0.000	0.000	0.000	0.000	0.000
Viscoelastica	CV (%)	4.117	15.524	−18.312	3.806	−9.663	0.353	NA	NA	NA	NA	NA	NA
Viscoelastica	MAD	0.843	0.643	0.000	0.758	0.007	0.013	NA	NA	NA	NA	NA	NA
maToni	Mitjana	23.244	−0.368	0.000	24.795	−0.039	3.462	NaN	NaN	NaN	NaN	NaN	NaN
maToni	SD	NA	NA	NA	NA	NA	NA	NA	NA	NA	NA	NA	NA
maToni	N	1.000	1.000	1.000	1.000	1.000	1.000	0.000	0.000	0.000	0.000	0.000	0.000
maToni	CV (%)	NA	NA	NA	NA	NA	NA	NA	NA	NA	NA	NA	NA
maToni	MAD	0.000	0.000	0.000	0.000	0.000	0.000	NA	NA	NA	NA	NA	NA
